# Cr-Doped Nanocrystalline TiO_2_-Cr_2_O_3_ Nanocomposites with p-p Heterojunction as a Stable Gas-Sensitive Material

**DOI:** 10.3390/ijms26020499

**Published:** 2025-01-09

**Authors:** Dmitriy Kuranov, Elizaveta Konstantinova, Anastasia Grebenkina, Alina Sagitova, Vadim Platonov, Sergei Polomoshnov, Marina Rumyantseva, Valeriy Krivetskiy

**Affiliations:** 1 Chemistry Department, Lomonosov Moscow State University, 119991 Moscow, Russia; nastya.greb@yandex.ru (A.G.); sagitova@inorg.chem.msu.ru (A.S.); agnes1992@yandex.ru (V.P.); roum@inorg.chem.msu.ru (M.R.); 2 Physics Department, Lomonosov Moscow State University, 119991 Moscow, Russia; liza35@mail.ru; 3 National Research University of Electronic Technology, Institute of Integrated Electronics and Microsystems, 124498 Moscow, Russia; psa@org.miet.ru; 4 Scientific-Manufacturing Complex Technological Centre, 124498 Moscow, Russia

**Keywords:** titanium dioxide, doping, pyrolysis, chromium, semiconductors, stability

## Abstract

Nanocrystalline TiO_2_ is a perspective semiconductor gas-sensing material due to its long-term stability of performance, but it is limited in application because of high electrical resistance. In this paper, a gas-sensing nanocomposite material with p-p heterojunction is introduced based on p-conducting Cr-doped TiO_2_ in combination with p-conducting Cr_2_O_3_. Materials were synthesized via a single-step flame spray pyrolysis (FSP) technique and comprehensively studied by X-ray diffraction (XRD), Brunauer–Emmett–Teller (BET) specific surface area analysis, transition electron microscopy (TEM), energy dispersive X-ray (EDX) spectroscopy, X-ray photoelectron spectroscopy (XPS), electron paramagnetic resonance (EPR), and Raman spectroscopy. Gas sensor performance in direct current (DC) mode was studied toward a number of gasses (H_2_, CO, CH_4_, NO_2_, H_2_S, NH_3_) as well as volatile organic compounds (VOCs) (acetone, methanol, and formaldehyde) in dry and humid conditions. The long-term stability of the obtained materials’ gas sensor performance was evaluated alongside with an ex situ study of structural evolution. High sensitivity toward oxygenated VOCs and a lower detection limit below ppm level with a limited influence of humidity were shown. The long-term gas sensor performance stability of the obtained materials and its connection to the defect structure of doped TiO_2_ is demonstrated.

## 1. Introduction

Metal oxide gas sensors are gaining interest lately due to the penetration of this technology to new market niches, particularly environmental monitoring [1,2,3], medical diagnostics [2,3,4], precision agriculture [5,6,7], safety in industrial [8], household [9,10], transportation [11,12,13], and food aspects [3,14], odor control [15,16,17], and others. Many of these new applications rely on a machine olfaction approach which consists of the utilization of machine learning algorithms, using data samples or datasets, obtained in controlled conditions, to build a model of individual gas sensor or sensor array response for further particular gas or gas mixture identification and/or quantification [18]. A weak spot of this powerful technique of gas analysis consists of the requirement of the long-term stability of gas sensor response, which currently hinders its widespread practical application [19,20,21,22,23]. In this regard, semiconductor titanium dioxide TiO_2_ attracts attention, as gas sensors based on it do not show signs of response degradation over time [24]. However, the application of this oxide in the gas sensor field meets difficulties due to high resistance and lower gas sensor responses, compared to the most often used SnO_2_. The n-type donor doping appeared to have limited effects due to the abundance of intrinsic electron-accepting defects, associated with Ti^3+^ cations [24]. The p-type doping by electron acceptor impurities is an alternative pathway to the improvement of the electrical and gas-sensing properties of nanocrystalline TiO_2_. The switch from the n-type to the p-type response of radio frequency (RF) sputtered TiO_2_ upon 4% at. doping with electron-accepting Cr(III) dopant was first tried almost three decades ago [25]. The increase in Cr content up to 10% and higher has been shown to result in prominent p-type behavior and in the formation of the Cr_2_Ti_2_O_7_ phase alongside with doped TiO_2_ during the sol–gel process [26]. The high doping concentrations of Cr (III) give rise to p-type conducting materials with much better conductance compared to intact n-type conducting TiO_2_, which is associated with the band-edge energy decrease, but the nature of p-type conductance remains unclear [27]. The Cr(III) dopant concentration, at which the transition from n-type to p-type conductance occurs, seems to be dependent on the synthesis technique and conditions and can reach 13% at. for RF-sputtering synthesis [28], while the utilization of the flame spray pyrolysis (FSP) route, known for the homogeneity of elements distribution in the end product of synthesis, gives rise to p-type conductance already at 1–5% at. Cr(III) loading [29,30]. The anodization procedure for obtaining TiO_2_ in the form of nanotubes allowed it to reach p-type conductance and sensor response at the 3% at. average content of Cr(III) dopant; however, the exact distribution of chromium ions between the surface and the inner volume of TiO_2_ nanotubes remained somewhat obscure [31,32,33]. The further work revealed the formation of a dopant energy level within the band gap, the position of which seemed to control the p-type conductivity and to be dependent on the Cr concentration [34]. According to the reported data, TiO_2_ always remains an n-type semiconductor with Cr doping below 1% at [35,36]. The p-type conducting TiO_2_ obtained via Cr(III) doping was shown to be sensitive toward hydrogen [25,37], nitrogen dioxide [26,27,38,39,40], ammonia [41], and ethanol [42], and some sensing characteristics toward CO and hydrocarbons are also available [39,43,44], and the oxygen content control with p-type Cr-doped TiO_2_ is possible too [26,45]. In most reported cases, the concentrations of gasses were too high compared to established safety limits or other levels, demanded by any exact application, due to a quite low sensor response of p-type TiO_2_. The use of p-p heterojunctions in the case of p-conducting semiconductor oxide materials has been recently shown as a fruitful approach for gas sensor response improvement [46,47,48].

In the present work, we report for the first time a successful attempt to create a gas-sensitive metal oxide composite material with p-p heterojunction on the basis of nanocrystalline p-conducting TiO_2_ and p-conducting Cr_2_O_3_. A series of Cr-doped TiO_2_ materials with a wide range of Cr(III) content from 2 to 40% mol both below and above the solubility limit (not higher than approx. 10% mol according to [29]) was synthesized. The flame spray pyrolysis (FSP) technique was utilized for synthesis to ensure the homogeneous distribution of elements. The obtained materials’ phase composition, morphology, and Cr chemical state and distribution across the TiO_2_ grains were thoroughly studied with a wide set of methods and instrumentation; gas sensor performance alongside with material structural aging by an ex situ approach was investigated in a comprehensive way.

## 2. Results and Discussion

**Figure 1:**
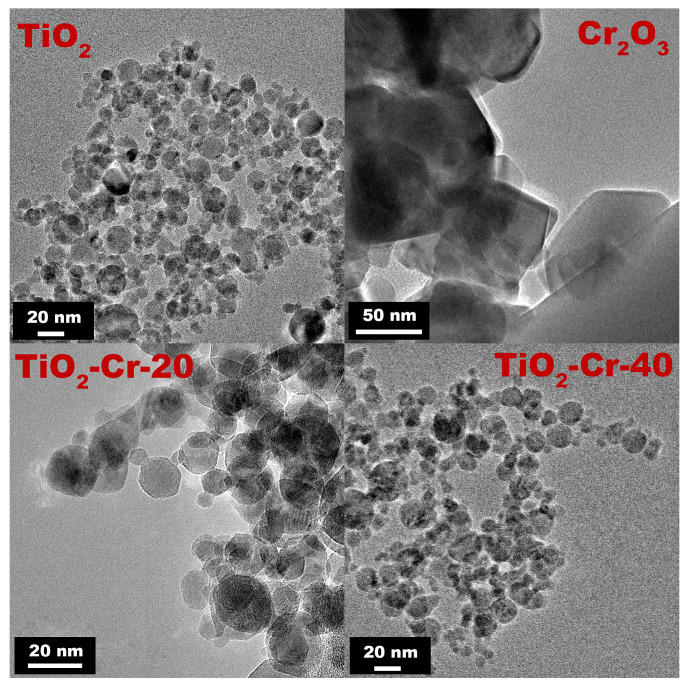
Bright-field TEM images of pure TiO_2_, Cr_2_O_3_, TiO_2_-Cr-20, and TiO_2_-Cr-40 samples.

The photographic images of synthesized materials are presented in Figure S1. Bright-field microphotographs of pure TiO_2_ and Cr_2_O_3,_ and also heavily doped materials TiO_2_-Cr-20 and TiO_2_-Cr-40 were obtained and are presented in Figure 1.

**Figure 2:**
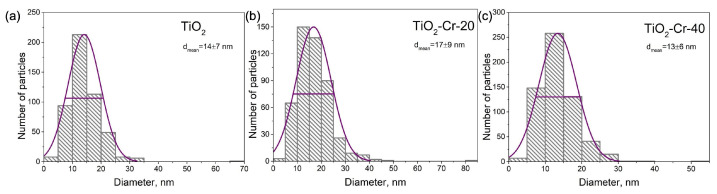
Histograms of normal particle distribution for (**a**) TiO_2_, (**b**) TiO_2_-Cr-20, and (**c**) TiO_2_-Cr-40 materials.

The particles of all TiO_2_-based materials are spherical in shape with a size mainly up to 20 nm, but there are also large ones, about 100 nm and higher. This represents normal distribution patterns with a small shoulder responsible for larger agglomerates (Figure 2). Each individual spherical particle is an agglomerate of smaller particles. The average particle size determined from TEM images is d_TEM_ (TiO_2_) = 14 ± 7 nm, d_TEM_ (TiO_2_-Cr-20) = 17 ± 9 nm, and d_TEM_ (TiO_2_-Cr-40) = 13 ± 6 nm. It has been shown that the particles of TiO_2_ doped materials become less like spheres with increasing Cr(III) content, and we notice the appearance of clear lines, edges, and corners. The TEM images of pure Cr_2_O_3_ reveal particles of greater size around 100 nm and bigger, which grow larger compared to TiO_2_ as a result of interplay between nucleation, agglomeration, surface growth, and coalescence processes [49]. Pure Cr_2_O_3_ consists of hexagonal particles with angles of 120° (Figure 1).

**Figure 3:**
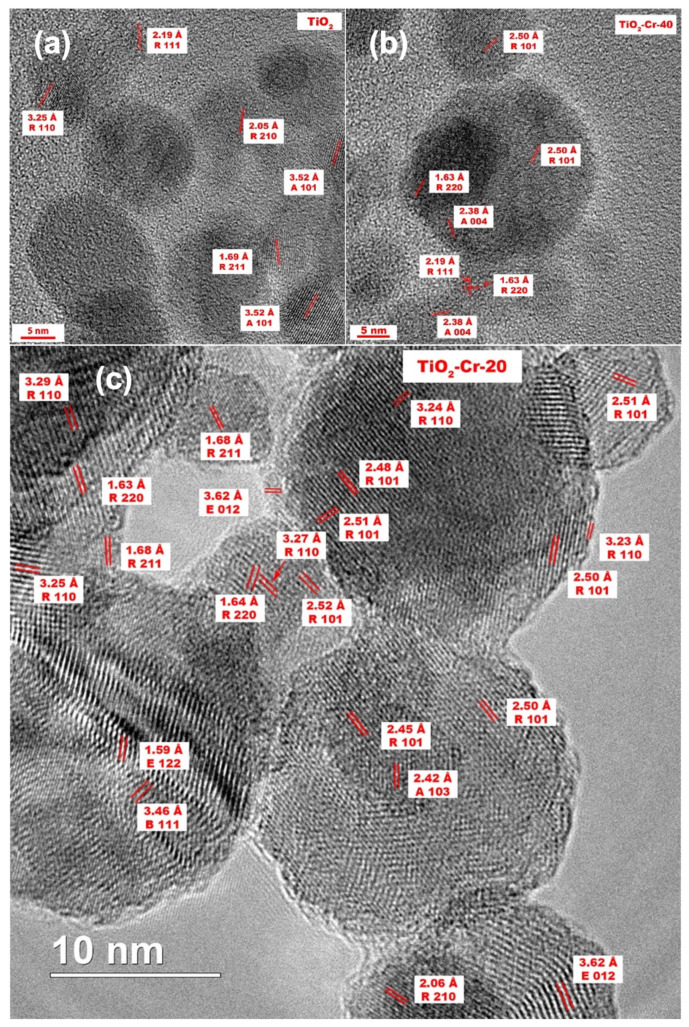
High-resolution TEM (HRTEM) images of (**a**) TiO_2_, (**b**) TiO_2_-Cr-40, and (**c**) TiO_2_-Cr-20 materials. A, R, and B in the white boxes are anatase, rutile, and brookite phases of TiO_2_ respectively_,_ and E is the eskolaite phase of Cr_2_O_3_, the 3-digit number next to phase designation are the Miller indices, the row above is the interplane distance in Angstroms. Red parallel lines indicate the crystallographic planes.

**Figure 4:**
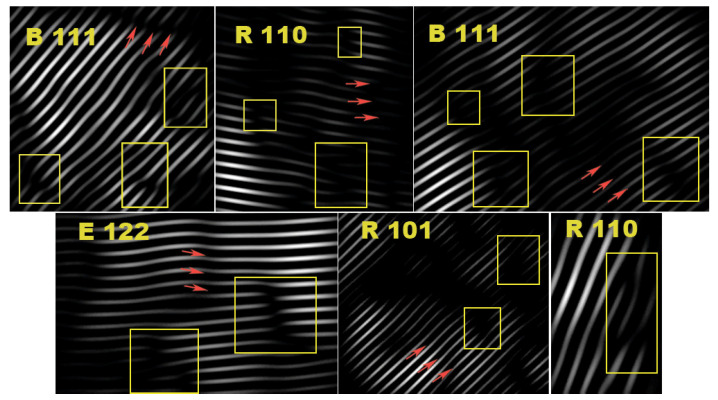
IFFT images of TiO_2_-Cr-20 phases. White lines are families of crystallographic planes, yellow frames indicate defects associated with the appearance of an extra half-plane or its absence, and red arrows indicate defects associated with shear as a result of doping. R, B are the rutile and brookite phases of TiO_2,_ and E is the eskolaite phase of Cr_2_O_3_, numbers next to phase designations are Miller indices of crystallographic planes.

The indexed electron diffraction pattern from a small accumulation of particles for a pure TiO_2_ contains concentric rings, characterizing the material as a physical mixture of anatase and rutile TiO_2_ phases (Figure S2). The patterns of TiO_2_-Cr-20 and TiO_2_-Cr-40 materials were characterized only by the reflections of the rutile phase, which is a consequence of its predominant content. High-resolution TEM images of TiO_2_ highlight the presence of anatase and rutile crystalline phases with corresponding interplanar spacings. Despite the fact that the electron diffraction patterns of highly doped materials do not contain anatase reflections, here we also observe some interplanar distances that characterize anatase and brookite (Figure 3). By analyzing several high-resolution photographs of TiO_2_ materials (Figure 3), it can be concluded that anatase and brookite particles are extremely rare. The formation of a Cr_2_O_3_ phase is observed at the grain boundaries of highly doped materials as evidenced by the corresponding interplanar distances. An analysis of the IFFT images of doped TiO_2_ (Figure 4) indicates a significant increase in the defectiveness during doping. Abundant lattice distortion in the form of curved planes is visible. Extended defects (edge and screw dislocations) are a consequence of the removal of one half-plane from the crystal lattice. Doping with Cr(III) leads to both the formation of defects and the fixation of existing ones. Being embedded in the crystal lattice, Cr strongly interacts with dislocation nuclei and their movement is slowed down and limited to short dashes. It is important that the number of dislocations does not depend on temperature, since their energy is too high.

**Figure 5:**
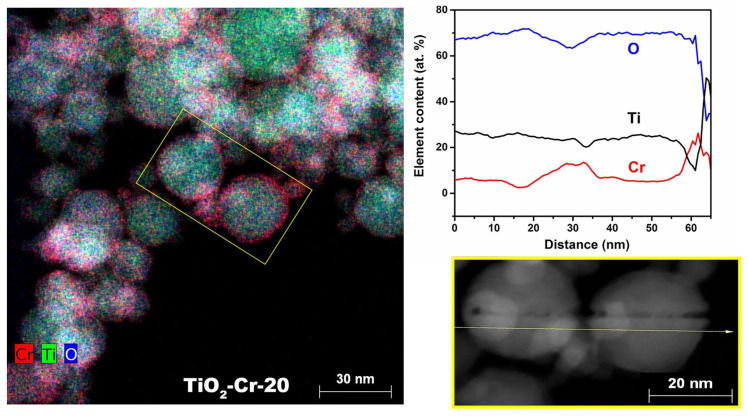
EDX map of Ti, Cr, O elements (**left**), and their content in certain places of yellow-boxed area (**right**) in TiO_2_-Cr-20 material.

Chromium, titanium, and oxygen are fairly evenly distributed in the TiO_2_-Cr-20 material (Figure 5), which correlates with the features of the synthesis method. However, there are areas of increased Cr concentration, especially at the edges of the particles as can be seen from the mapping «along the line» in Figure 5. It is shown that within individual particles the modifier is distributed evenly; however, near the boundaries, jumps in content are observed, indicating the formation of a Cr_2_O_3_ impurity phase, which is especially clearly observed at the coordinates of 33 and 58 nm (Figure 5). The energy-dispersive X-ray study of doped materials confirms the uneven Cr distribution (see Figure S3). Chromium content in the middle of a randomly selected particle was lower than the specified value (7 at.%, with a given 20 at.%). Hereby, according to the obtained element maps, areas with an increased Cr content near the particle boundaries are observed, which is associated with the formation of Cr_2_O_3_ due to the limited solubility of Cr^3+^ in TiO_2_ phases, as well as the formation of Cr^6+^, whose too small ionic radius does not allow it to participate in the formation of substitutional solid solutions, r_i_$\left({Cr}_{VI}^{6+}\right)$ = 0.44 Å [50].

**Figure 6:**
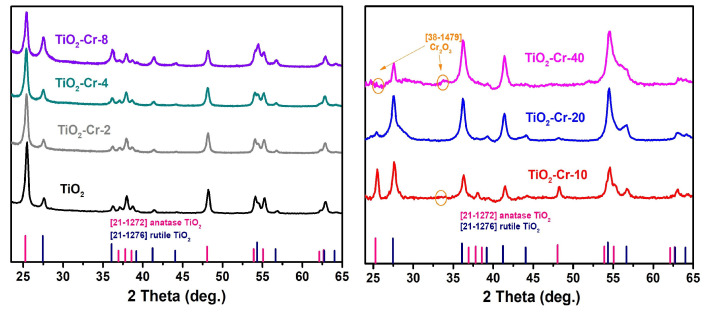
The X-ray diffraction patterns for TiO_2_-Cr materials.

The X-ray diffraction patterns (Figure 6) of TiO_2_ powder samples with a Cr(III) content of up to 8 mol.%, inclusive, are characterized by the presence of reflections only of the anatase and rutile TiO_2_ phases, while the diffraction patterns of samples with a higher Cr(III) content have additional reflections associated with the presence of the eskolaite phase Cr_2_O_3_ (ICDD 38-1479, Figure S4), which is due to the limited solubility of chromium in TiO_2_ phases. The composition and microstructure parameters of synthesized materials are presented in Table 1. It follows that with an increase in the content of Cr(III) in the materials, there occurs (i) a monotonic increase in the proportion of the thermodynamically stable rutile phase and (ii) a tendency to increase in the specific surface area and an accompanying decrease in the degree of agglomeration in accordance with the previously reported study on Cr-doped TiO_2_ nanopowders [51]. This anatase to rutile transformation may be dependent on the materials’ morphology and synthesis procedure as the anatase phase stabilization upon Cr doping has been reported previously for thin-film samples as well [32].

The parameters of the unit cells of the anatase and rutile phases were refined using several of the most intense reflections that do not overlap with the reflections from the Ge standard (ICDD 4-545). The dependences of changes in the values of some interplanar spaces are presented in Figure S5. It follows that the introduction of Cr(III) into the Ti sublattice leads to an increase in the parameters of unit cells, since in an octahedral oxygen environment ${Cr}_{VI}^{3^{+}}$ has a larger ionic radius (r_i_ = 0.615 Å) than ${Ti}_{VI}^{4^{+}}$ (r_i_ = 0.605 Å). The obtained values of the unit cell parameters (Figure S6) of the anatase and rutile TiO_2_ phases are compared with the values calculated using Vegard’s law [52] and correlate well with them. The plateau in the dependences of unit cell parameters and interplanar distances is associated with the achievement of the Cr(III) solubility limit in the TiO_2_ phases (Figures S5 and S6). The decrease in the calculated values of the tensile microstresses of the anatase phase lattice with increasing chromium content is nontrivial, since, as noted above, the radius of the dopant is higher (Table 1). For the rutile phase, the dependence has a non-monotonic character—first, a decrease and then a slight increase. It is possible that other defects are formed that lead to lattice compression, as well as the formation of chromium in a high oxidation state.

As a result, it can be stated that the increase in Cr-content in the FSP synthesis precursor solution gives rise to a composite material with nanosized grains of Cr-doped TiO_2_ with the surface decorated with the segregated nanocrystalline Cr_2_O_3_ phase.

**Figure 7:**
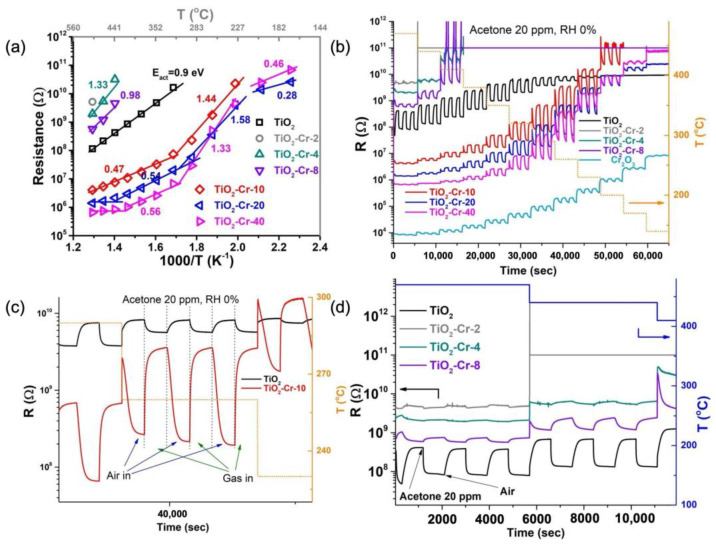
(**a**) R(T) dependence of materials in dry clean air. (**b**) Resistance transients of Cr-doped TiO_2_-based materials under gas phase composition and temperature changes. (**c**) Detailed resistance transients of pure TiO_2_ and TiO_2_-Cr-10 materials in the same conditions. (**d**) Detailed resistance transients of pure TiO_2_ and lightly Cr-loaded materials in the same conditions.

A composite nature of the obtained materials is manifested in the changes in their electrical resistance along with the increase in the Cr content in the synthesized samples. The dependences of electrical resistance on working temperature (T) for thick TiO_2_-based films deposited on Al_2_O_3_ substrates in the dry clean air media were obtained in the temperature range of 140–500 °C (Figure 7a).

The activation nature of the conductivity is confirmed by its linear type in logR versus 1/T coordinates. Pure TiO_2_ is an n-type semiconductor, where the main charge carriers are conduction electrons, thus the resulting linear dependence characterizes its own conductivity. Small proportions of Cr (2–8%) lead to a sharp increase in the resistance of materials, which is associated with the formation of substitutional solid solutions. Doping with Cr(III) leads not only to a change in the concentration of oxygen vacancies in materials but also to the formation of conduction holes. After reaching the chromium content up to 2%, intrinsic conduction electrons are no longer the only charge carriers and can recombine with conduction holes according to Equations (1) and (2).

(1) $${\mathrm{Cr}}_{2}O_{3}\;\overset{{TiO}_{2}}{\to }2{Cr}_{Ti}^{\prime }+V_{O}^{x}+3O_{O}^{x}+2h^{\cdot }$$

(2) $$e^{\prime }+h^{\cdot }\overset{{TiO}_{2}}{\to }\mathrm{nil}$$

where ${Cr}_{Ti}^{\prime }$ is the chromium (III) on titanium (IV) position; $V_{O}^{x}$ is neutral oxygen vacancy; $O_{O}^{x}$ is oxygen in its position in the TiO_2_ lattice (Kröger–Wink notation).

The highest electrical resistance has been detected for the TiO_2_ with 2% mol doping (TiO_2_-Cr-2) which decreased below 10 GOhm only at a 500 °C working temperature, while at lower temperatures, it was beyond the measurement limit of the used electrical circuit. It appears that Cr 2% mol content of doping impurity corresponds to an almost complete recombination of charge carriers. An increase in Cr content up to 4 and 8% mol leads to a slight increase in the conductance of thick films, which appeared to be a p-type conductance as is shown further. This p-type conductance is attributed to the domination of hole charge carriers of Cr-doped TiO_2_. Interestingly, a further increase in Cr(III) content from 10 up to 40% mol results in a sharp decrease in the resistance of the materials. This abrupt change indicates a switch from Cr-doped TiO_2_ conductance to a conductance of the Cr_2_O_3_ phase, continuously covering the Cr-doped TiO_2_ grains and agglomerates, as is shown by TEM and EDX mapping.

The mode of the atmosphere composition changing in the gas flow chamber, the working temperature of the sensors, as well as the associated effects on the resistance of sensitive elements, is conveyed by Figure 7b,c. It was shown that doping with Cr(III) led to a monotonic decrease in the resistance of materials from 10^7^–10^8^ Ω for the TiO_2_ material down to 10^3^ Ω for the TiO_2_-Cr-40 material at a temperature of 500 °C. Pure TiO_2_ demonstrated n-type conductivity while Cr(III)-doped materials, as well as pure Cr_2_O_3_, demonstrated p-type conductivity, as evidenced by the type of R(T) dependence during the change from clean air to a reducing (acetone) gas containing atmosphere flow with zero relative humidity (RH = 0%).

The sensor response has been calculated according to Formula (3):

S = |σ_gas_ − σ_air_|/σ_air_ (3)

where σ_gas_ and σ_air_ are the conductances of sensing elements in the presence of detected gas and in the flow of clean air, respectively.

**Figure 8:**
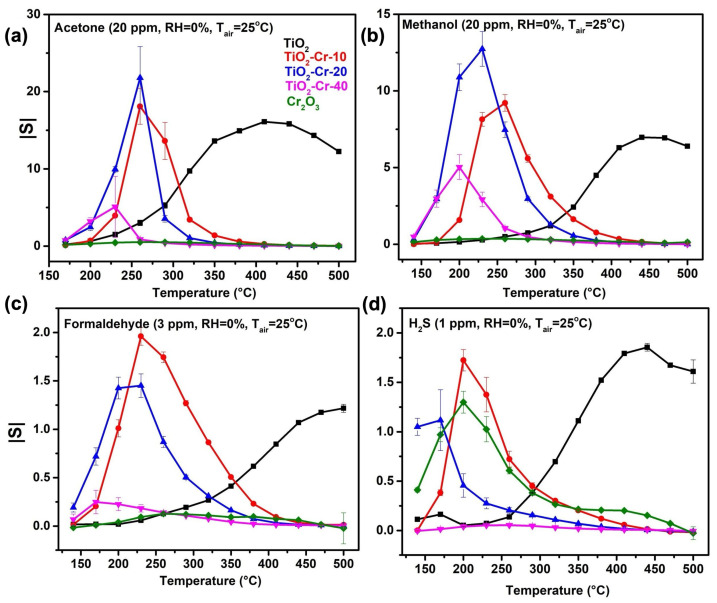
The temperature dependencies of the sensor signal of p-TiO_2_-Cr_2_O_3_ composites toward VOCs ((**a**) acetone, (**b**) methanol, (**c**) formaldehyde) and (**d**) hydrogen sulfide compared to the gas sensor responses of pure n-TiO_2_ and Cr_2_O_3_. The modulus of sensor response is given for the sake of easier comparison, while all Cr-containing materials behave as p-type semiconductors, and pure TiO_2_ shows n-type behavior.

The S(T) dependences toward acetone, as well as other gasses and volatile organic compounds, all of which behave as reducing gasses, i.e., the reducing resistance of pure TiO_2_ and the increasing resistance of TiO_2_-Cr composites, are shown in Figure 8. It can be seen that pure TiO_2_ and composites exhibit the greatest sensitivity to oxygen-containing volatile organic compounds (VOCs), acetone (20 pm), formaldehyde (3 ppm), and methanol (20 ppm), significantly outperforming Cr_2_O_3_. Pure chromium oxide showed a relatively high signal, comparable to pure TiO_2_ and composites, only to H_2_S (1 ppm). A switch from n-type to p-type conductivity led to the disappearance of sensitivity toward NO_2_ in the case of composite materials (Figure S7). This indicates an inability of this molecule to chemisorb on the surface of the metal oxides according to Equation (4) due to the absence of free electrons in the conduction band of semiconductors:

(4) $${NO}_{2}+e^{-}↔{NO}_{2ads}^{-}$$

Another consequence of the absence of free electrons in the composite system is the inability of highly reactive chemisorbed oxygen species to form on the surface of metal oxide according to Equation (5):

(5) $$O_{2}+e^{-}↔O_{2ads}^{-}$$

It means that the oxidation of VOCs on the materials’ surface, resulting in the observed gas sensor response, processes mostly via the Mars–van Krevelen mechanism and consists of the participation of metal oxide lattice oxygen ions in the oxidation of adsorbed molecules with the further compensation of oxygen loss from ambient air [24]. This mechanism is illustrated by Equations (6)–(9):

(6) $${CH}_{3}CO{CH}_{3ads}+{8O}_{lat}^{2-}\to {3CO}_{2}↑+3H_{2}O↑+16e^{-}$$

(7) $$O_{2air}+e^{-}↔O_{2ads}^{-}$$

(8) $$O_{2ads}^{-}+e^{-}↔{2O}_{ads}^{-}$$

(9) $$O_{ads}^{-}+e^{-}↔O_{lat}^{2-}$$

**Figure 9:**
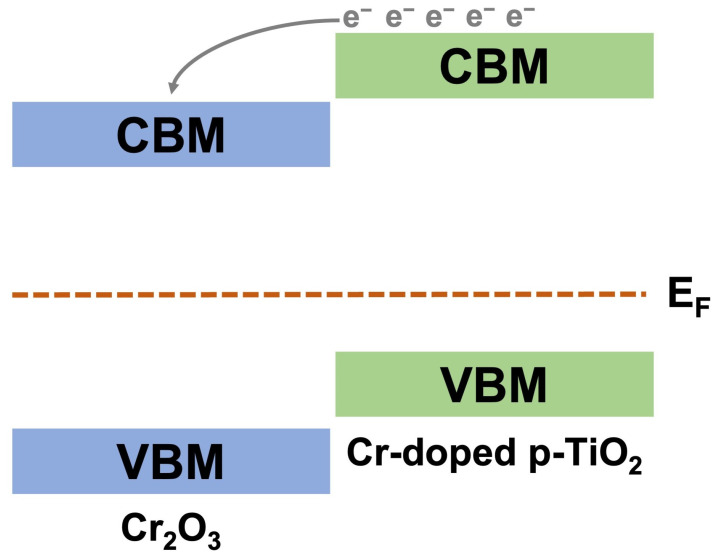
Schematic electronic band diagram of p-p heterojunction during the gas-sensing process.

The free electrons forming during the course of chemical oxidation via Equation (6) recombine with conduction holes resulting in the according resistance increase. The response effect is much more pronounced in the case of composite materials as the additional free electrons migrate to the conduction band of the Cr_2_O_3_ phase through heterojunction with p-conducting Cr-doped TiO_2_ via the scheme, depicted in Figure 9.

The absence of chemically active chemisorbed oxygen species on the surface of metal oxide nanocomposites is the reason why relatively chemically inert hydrocarbons, as well as simple molecules with weak adsorption on the metal oxide surface such as H_2_ (50 ppm) and CO (20 ppm), do not give rise to distinct sensor signals (Figure S7).

A significant decrease in the temperature of maximum sensor response toward VOCs in the case of TiO_2_-Cr composite materials—down to 200–250 °C compared to 400–450 °C in the case of pure TiO_2_—is observed. This low working temperature range corresponds to the region of the maximum sensitivity of the Cr_2_O_3_ conductance to the concentration of thermally excited free charge carriers (holes) according to Figure 7a,b. Above 300 °C, the resistance of Cr_2_O_3_ is not very sensitive to hole concentration, as a different mechanism of conductivity—small-polaron hopping—is realized [53].

The optimal Cr content for gas sensor response maximization is in the 10–20% mol range, while its increase of up to 40% mol leads to a distinct decrease. This phenomenon should be attributed to the low sensor sensitivity of pure Cr_2_O_3_, which at this loading seems to become a dominating phase in the chemical interaction with the gas phase and is separated from the surface of Cr-doped TiO_2_, thus not participating in the p-p heterojunction formation.

**Figure 10:**
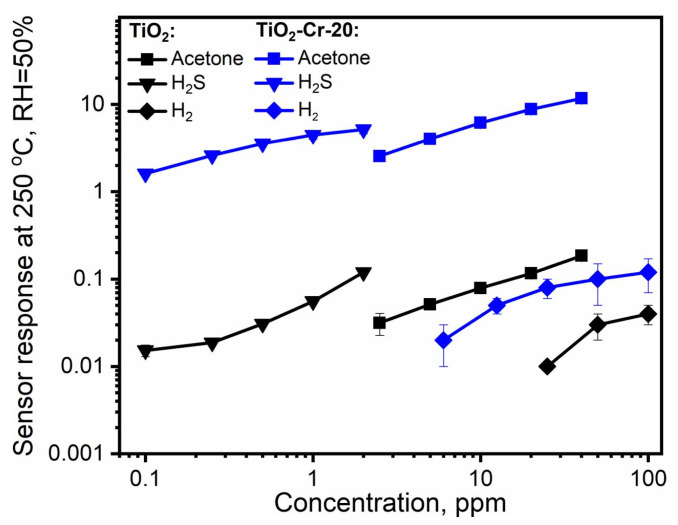
Cross-sensitivity of obtained materials toward gasses of different chemical nature at the operating temperature optimal for VOCs detection in humid conditions. The modulus of sensor response is given for the sake of easier comparison, while all Cr-containing materials behave as p-type semiconductors and pure TiO_2_ has shown n-type behavior.

The cross-sensitivity of the obtained materials is given in Figure 10. It clearly shows the improved selectivity of responses toward VOCs and H_2_S in the case of composites compared to pure TiO_2_; however, the discrimination between H_2_S and VOCs as well as the different types of oxygenated VOCs may be difficult in the isothermal operation mode of gas sensors and without any kind of signal processing.

**Figure 11:**
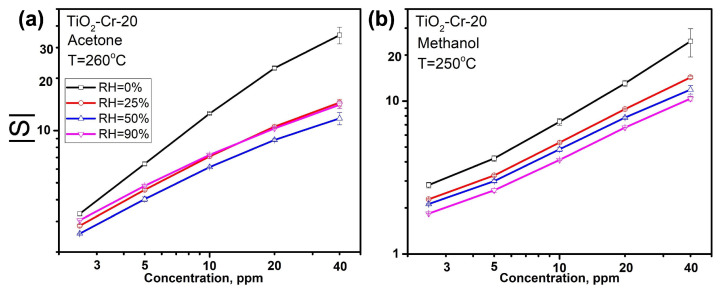
Concentration dependencies of the sensor signal of TiO_2_-Cr-20 material at different humidity values towards (**a**) acetone and (**b**) methanol. The modulus of sensor response is given for the sake of easier comparison, while all Cr-containing materials behave as p-type semiconductors, and pure TiO_2_ shows n-type behavior.

The concentration dependences of the sensor response for TiO_2_-Cr-20 material, which shows the highest response, toward acetone and methanol at different values of relative humidity are shown in Figure 11. The curves are linearized in double logarithmic coordinates, from which the average minimum detectable concentration (C_min_) values were calculated, which amounted to 0.61 and 0.85 ppm for acetone and methanol gasses, respectively. It is shown that the sensors function acceptably even at high humidity values, making it possible to detect these analyte gasses at sub-ppm concentrations.

**Figure 12:**
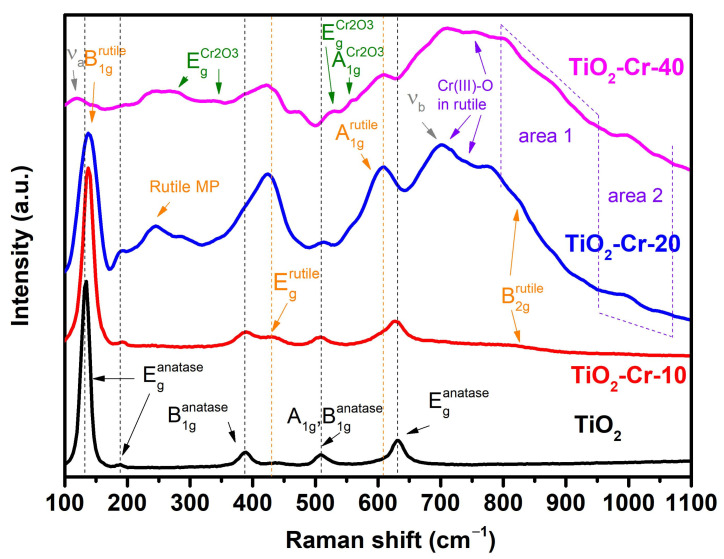
Raman spectra for TiO_2_, TiO_2_-Cr-10, TiO_2_-Cr-20, and TiO_2_-Cr-40 materials.

The nature of the high sensor response of Cr-doped p-type TiO_2_–Cr_2_O_3_ nanocomposites toward oxygen-containing VOCs can be clarified through additional studies of the surface structure and chemical state. The presence of an additional crystalline phase of Cr_2_O_3_ in highly doped materials (at a given Cr content of more than 8 mol%) was clarified using Raman spectroscopy (see Figure 12). It is known that in the presence of a significant fraction of the amorphous phase in the material, a halo can appear in the Raman spectra and the characteristic modes of the phases can disappear. In the Raman spectrum of the TiO_2_-Cr-40 material, this is most clearly manifested by the appearance of an extensive halo from the amorphous phase and the almost complete disappearance of the B_1g_ mode of the rutile phase around 140 cm^−1^.

The spectrum of pure TiO_2_ contains the Raman modes characteristic for the anatase and rutile phases [54,55,56,57].

The spectrum of the TiO_2_-Cr-10 material exhibits a blue shift in the anatase phase mode (~137 cm^−1^) and its broadening, as well as a red shift in the anatase phase mode E_g_ (~627 cm^−1^), associated with doping and a decrease in grain size. The appearance of the B_2g_ (826 cm^–1^) rutile phase is associated with an increase in the proportion of this phase in the material. The spectra of TiO_2_-Cr-20 and TiO_2_-Cr-40 materials are complicated by an increase in the intensity of the E_g_ and A_1g_ modes of the rutile phase, as well as the appearance of the characteristic modes of the Cr_2_O_3_ phase (3E_g_: 282, 347, and 527 cm^−1^ and A_g_: 556 cm^−1^) [58,59,60]. An observed second-order scattering (multiphonon scattering, MP), most noticeable at ~237 cm^−1^ (E_g_), is also considered to be a characteristic Raman peak of the rutile phase [61]. It was reported [62] that such changes in the Raman spectrum of the rutile TiO_2_ phase are observed only at high dopant concentrations or very small, less than 25 nm, particle sizes. Cr-doped samples have broader peaks, which are also characterized by a blue shift B_1g_ and a red shift E_g_. New contributions appearing at 110–120 cm^−1^ and 700 cm^−1^ are marked as ν_a_ and ν_b_, respectively, in the spectra of TiO_2_-Cr-20 and TiO_2_-Cr-40. The contribution of ν_a_ can be estimated as a shoulder next to the B_1g_ mode, which is a consequence of a decrease in the grain size of the rutile phase and/or the amorphization of the surface. The enhancement of the ν_a_ and ν_b_ modes in highly doped materials, together with the observation of the B_2g_ mode, is explained in [63,64] as a violation of the Raman selection rules. Selection rules can be violated due to the contribution of disorder, as is observed, for example, in a material with an amorphous surface. The $B_{1g}^{rutile}$ mode, which is absent for the material of pure TiO_2_, appears for the material of TiO_2_-Cr-10 (FWHM = 19.35 cm^−1^) and undergoes a significant broadening for the material of TiO_2_-Cr-20 (FWHM = 32.48 cm^−1^), which is associated with the formation of a significant number of defects and surface amorphization. An alternative explanation for this broadening may be that this peak is a combination of the two adjacent modes of intense $E_{g}^{anatase}$ and weak $B_{1g}^{rutile}$.

As mentioned in [65], the new mode at 750 cm^−1^ is presumably attributed to the local ν(Cr^III^-O) vibration in the TiO_2_ lattice and is possibly subject to the Raman resonance effect (λ = 532 nm of the laser falls in the optical absorption region of chromium particles). Reference Cr(III) compounds exhibit Raman modes in the region of 500–600 cm^−1^ due to their lower oxidation state compared to Cr^(VI)^ compounds, which exhibit Raman modes in the region of 800–1100 cm^−1^ [66]. Region 1 (800–950 cm^−1^) reflects the presence of the antisymmetric and symmetric Cr-O-Cr stretching vibrations of terminal CrO_3_ groups [66]. Region 2 (950–1100 cm^−1^), in turn, consists of the symmetric and antisymmetric stretching vibrations of the CrO_2_ fragment in a tri- or tetramer [67]. In [68], two bands were observed for TiO_2_-Cr materials at 995 and 1005 cm^−1^. The first band is associated with the vibration of the Cr=O bond on the surface of TiO_2_. The second band corresponds to the same, but under dehydrated conditions caused by laser heating. Thus, the Raman spectroscopy study reveals that highly Cr-loaded materials represent nanocomposites that contain abundant Cr_2_O_3_ crystalline phase, dispersed over the surface of Cr-doped TiO_2_ grains and their agglomerates. Particularly interesting is the presence of chromium in a highly oxidized form Cr^6+^ on the surface of materials, which can act as an adsorption site with high Lewis acidity, promoting the adsorption of VOCs [69,70].

**Figure 13:**
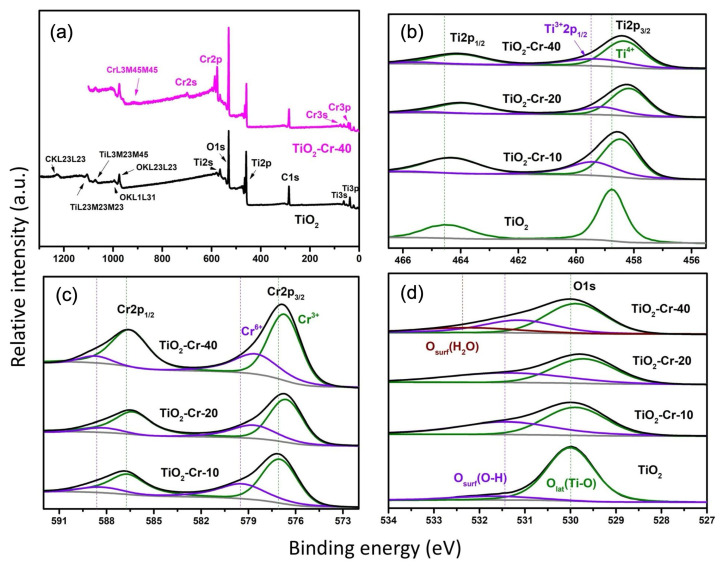
(**a**) Survey and detailed XPS spectra in the energy ranges of (**b**) Ti 2p, (**c**) Cr 2p and (**d**) O 1s signals for TiO_2_, TiO_2_-Cr-10, TiO_2_-Cr-20, and TiO_2_-Cr-40 materials. Dotted lines are given for the sake of clarity of the photoemission spectra energy shifts upon increase of Cr content.

The study of the electronic state of elements in pure and Cr(III)-doped TiO_2_ was carried out by the XPS and EPR spectroscopy methods. A survey and detailed XPS spectra of pure and highly Cr-doped TiO_2_ materials are shown in Figure 13.

Titanium is expectedly present in the Ti^4+^ form of the charge form in pure TiO_2_ material (doublet, Ti2p_3/2_ 458.75 eV, Ti2p_1/2_ 464.45 eV, multiplet splitting 5.7 eV) [71,72]. Two singlet components can be distinguished in the oxygen spectrum: a peak at 530.0 eV, corresponding to the presence of lattice oxygen TiO_2_ (O_latt_._),_ and a peak at 531.5 eV, which can be attributed to the various forms of chemisorbed oxygen and surface hydroxyl groups (O_surf_.) [73,74]. The value of Δ(Ti^4+^-O_latt_.) undergoes an increase with increasing Cr content in materials from 71.2 eV (for TiO_2_) to 71.6 eV (for TiO_2_-Cr-40), which is reflected in Table 2, which is a consequence of a change in the charge state of Ti [75,76]. The detailed spectrum of Cr (TiO_2_-Cr-10) consists of two doublets Cr2p_3/2_ and Cr2p_1/2_, with the positions of the main peaks at 577.01 and 579.39 eV, respectively, which corresponds to the presence of chromium in two charged states of Cr^3+^ and Cr^6+^ (the splitting values were 9.69 and 8.95 eV, respectively) [77,78,79]. We estimate the ratio of Cr^3+^ to Cr^6+^ to be 3:2, with a tendency toward a slight increase in the proportion of Cr^3+^ with increasing Cr content in doped materials. The value of Δ(Cr^3+^-O_latt_.) for Cr-doped materials undergoes a monotonic decrease from 47.1 eV (TiO_2_-Cr-10) to 46.8 eV (TiO_2_-Cr-40), which also indicates partial charge transfer [75]. The deconvolution of the detailed spectrum of the Ti2p region of the doped materials reveals an additional component, which the authors of [80,81,82] associate with the presence of an element in the charge form Ti^3+^ (Ti2p_1/2_) next to the oxygen vacancy. In addition, all photoemission spectra of elements undergo a shift, which is observed with the increase in Cr content in materials, as well as a change in the spin–orbit splitting of the detailed spectra of Cr and Ti, which indicates a change in the charge state of the elements [75,83,84]. However, the appearance of an additional component may also be due to the fact that the Ti-O-Ti bonds in the TiO_2_ lattice are partially replaced by Ti-O-Cr during doping. A pattern has been established of an increase in the content of surface oxygen in the materials with increasing Cr content, and the peak of surface oxygen for the TiO_2_-Cr-40 material can also be decomposed into its components: hydroxyl oxygen and adsorbed water [84]. The additional component O_(H2O)_ is characterized by an increased binding energy of 532.3 eV. Despite the fact that XPS is a semi-quantitative determination method, we assessed the Cr content on the surface of the materials. The calculated values of the Cr mole fraction correlate well with the value specified during synthesis for the TiO_2_-Cr-10 material and turned out to be greatly underestimated for the TiO_2_-Cr-20 and TiO_2_-Cr-40 materials (Table 2). The most likely reason is the uneven distribution of Cr between the bulk and surface of the material.

**Figure 14:**
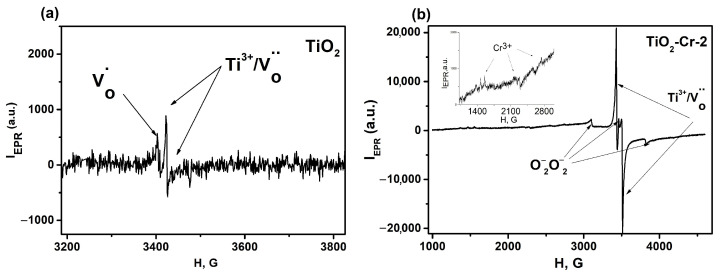
EPR spectra of (**a**) TiO_2_ and (**b**) TiO_2_-Cr-2 materials.

The informative EPR spectra of pure TiO_2_ and TiO_2_-Cr-2 materials were obtained. For the Cr-doped TiO_2_-Cr_2_O_3_ nanocomposites (TiO_2_-Cr-20 and TiO_2_-Cr-40 samples), EPR spectra were found to be uninformative, since an abundance of Cr^3+^ ions leads to a strong dipole–dipole interaction, as a result of which the lines are greatly broadened and only their envelope is visible. (Supplementary part, Figure S8 g = 1.9712). EPR allows the distinguishing of signals from the different types of oxygen vacancies because the centers localizing the electron density in the spectra are shifted toward lower magnetic field values. Oxygen vacancies are detected in an undoped TiO_2_ sample (Ti^3+^/V_o_˙˙ centers with g-factors: g = 1.9878, g = 1.9612) as well as the oxygen vacancies that captured an electron (V_o_ + e′ with g-factors: g = 2.0012, g = 1.9997) and designated according to Kröger-Vink notation as V_o_˙ [85,86,87], Figure 14.

The concentration of the defects in Ti^3+^/V_O_ was 1.2⋅10^15^ g^−1^, and V_O_ + e’- 7.2⋅10^14^ g^−1^ in the TiO_2_ sample. The paramagnetic centers of various nature were found in the TiO_2_-Cr-2 sample. Firstly, Cr^3+^ ions in an insignificant concentration are shown on an enlarged scale in the inset in the field region of 1000–3000 Gauss. Secondly, Ti^3+^/V_O_ oxygen vacancies that do not localize electrons (g_1_ = 1.9798, g_2_ = 1.9605) registered in the magnetic field range of 3400–3600 Gauss. The concentration of Ti^3+^/V_O_ oxygen vacancies, formed according to Equation (1), for the TiO_2_-Cr-2 material was 3.4⋅10^18^ g^−1^, which is more than three orders of magnitude higher than in the undoped material (1.2⋅10^15^ g^−1^). Thirdly, we also discovered an EPR signal in the magnetic field range of 3000–4000 Gauss, consisting of three lines, which, to the best of our knowledge, has never been reported before. We assume that superoxide biradicals with a spin equal to one are responsible for it. It can be hypothesized that two species of chemisorbed oxygen $O_{2}^{-}$ located on the same adsorption center, e.g., the surface Cr(VI) cation, led to the appearance of such a signal in the EPR spectra. Alternatively, it can be attributed to another radical molecular uncharged oxygen O_2_, since it also has a spin equal to one due to the presence of two unpaired electrons; however, this form of oxygen can be detected on the spectrum in the regions of magnetic field strength of about 30,000 G. It is important to note that the EPR spectrum of the TiO_2_-Cr-2 material has a shoulder in the spectral region around H = 3480 G. Chromium ions with intermediate values of charge states can be detected exactly in this region, which can be interpreted as Cr^5+^ [88] (as a superposition of two charged forms of Cr^3+^ and Cr^6+^) [69].

Concluding this section, it is important to note the formation of Cr (VI) cations with high Lewis acidity both on the Cr-doped TiO_2_ surface even with a low doping level, as well as in the case of Cr-doped TiO_2_-Cr_2_O_3_ nanocomposites. These highly acidic cations facilitate the strong chemisorption of oxygenated VOCs promoting their further oxidation via the Mars–van Krevelen route and the enhancement of the gas sensor response.

### Long-Term Gas Sensor Performance

**Figure 15:**
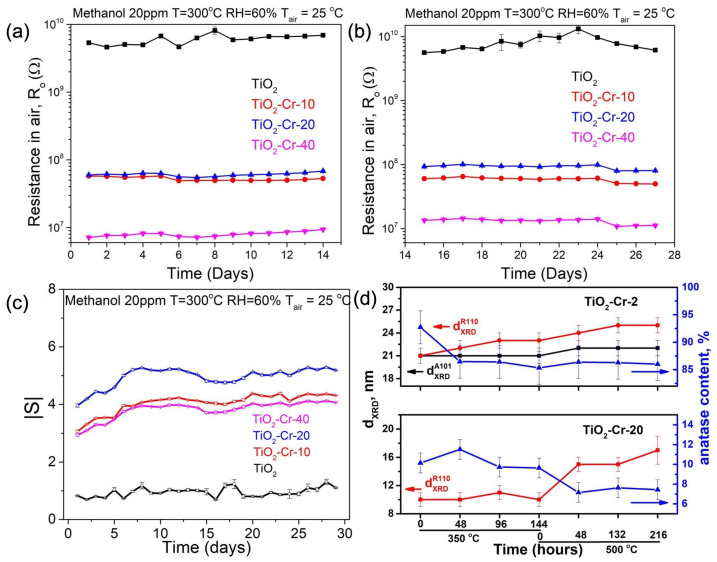
(**a**,**b**) Resistance in air and (**c**) sensor response toward 20 ppm of methanol obtained in the long-term continuous experiment (T = 300 °C, RH = 60%, T_air_ = 25 °C). (**d**) Evolution of TiO_2_-Cr materials during a sensor-testing simulation experiment.

Methanol (20 ppm) in humid air (RH = 60%) was chosen as an analyte gas to study the stability of the sensor characteristics of materials during long-term operation. The mode of alternation of the atmosphere composition in the sensor chamber consisted of the 900-second period of gas flow through the sensor chamber, which was followed by a 1800 s period of clean humid air flow to ensure the complete removal of methanol traces and possible reaction products from the sensing material surface. It is shown (Figure 15a–c) that the basic resistivity of doped materials in clean air media did not undergo strong changes during the experiment. The experiment lasted for 27 days with a 10-day pause in the middle. Pure TiO_2_ demonstrated a fairly stable gas sensor response over the whole experiment as was expected from the previous study, while nanocomposite materials took one week to reach stable performance. Interestingly, during this week, the sensor response was improving. The response times of TiO_2_-Cr-20 and TiO_2_-Cr-40 materials also improved during the long-term operation (Table 3), while response and recovery did not change their pace in the case of the pure TiO_2_ gas sensor.

A simulation of the sensing experiment in a tube furnace was performed to determine the cause of the changes in the long-term characteristics of the materials.

An ex situ study using the X-ray diffraction method for powder materials that were annealed under conditions simulating the sensor experiment (temperature, gas phase composition, duration of exposure, and frequency of gas phase composition change; see Experimental Section) was carried out for TiO_2_-Cr-2 and TiO_2_-Cr-20 materials. A change in the ratio of anatase/rutile phases, as well as the size of TiO_2_ crystallites depending on the experimental conditions is shown in Figure 15d. The calculation was carried out in accordance with the diffractograms taken after each stage of the sensor experiment simulation. It is shown that for both materials an increase in the grain size of the phases was observed due to sintering. Already at a temperature of 350 °C, the TiO_2_-Cr-2 material undergoes a polymorphic transition under gas sensor experiment conditions, while for the TiO_2_-Cr-20 material, the mass fraction of the anatase phase remains unchanged up to an increase in temperature to 500 °C.

**Figure 16:**
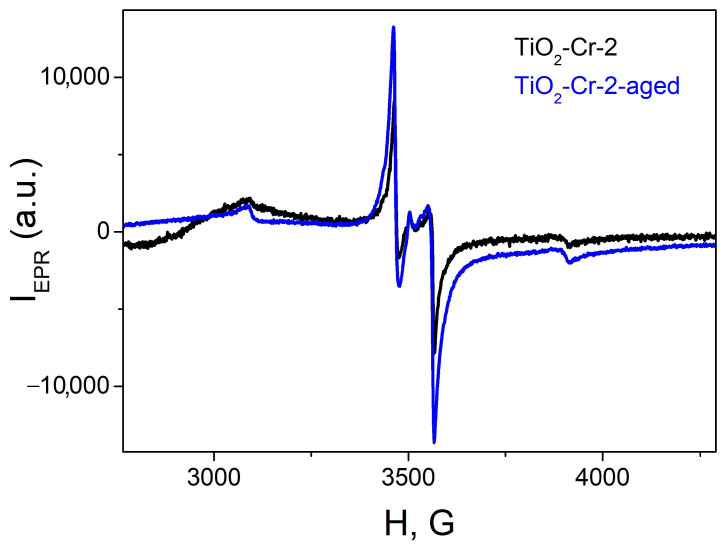
EPR spectra of as prepared and aged TiO_2_-Cr-2 material according to protocol of long-term gas sensor measurements.

The EPR study of the TiO_2_-Cr-2 sample before and after aging in long-term gas sensor experiment conditions has shown an increase in the concentration of neutral oxygen vacancies (see Figure 16).

The initial concentration of 3.4⋅10^18^ g^−1^ for neutral oxygen vacancies reached the value of 5⋅10^18^ g^−1^ after aging. This increase is attributed to the slow process of the incorporation of Cr (III) ions into the TiO_2_ lattice during the materials’ operation as a sensing element at an elevated temperature. This means the presence of the surface-segregated Cr(III) amorphous phase even for lightly doped TiO_2_, which is detectable only by EPR measurements. Secondly, this slow substitutional defect formation gradually affects the charge carrier concentration, and for Cr-doped TiO_2_-Cr_2_O_3_ composites it is responsible for observed gas sensor response improvement during the first week of operation (Figure 15). Some degree of nanocomposite gas sensor response improvement should also be attributed to the improvement of the intergrain boundary and the enhancement of contact between the p-conducting phases as this sintering is also happening according to the ex situ XRD study (Figure 15d).

## 3. Materials and Methods

The synthesis of semiconductor nanocrystalline TiO_2_ materials was carried out by flame spray pyrolysis (FSP). A precursor titanium (IV) triisostearoyl isopropoxide (Gelest Inc., Morrisville, PA, USA) purity 90%, titanium content 8.3–9.1%) was chosen as a source of titanium. A series of solutions (solvent–toluene, purity 99.5%, Ekos-1, Moscow, Russia) containing chromium from 0 to 40 mol percent (by cations) was prepared in a glovebox to protect precursors from hydrolysis, and chromium (III) 2-ethylhexanoate (70% in mineral spirits, Gelest Inc., Morrisville, PA, USA) was used as a source of chromium. Prepared solutions with C(Ti) = 0.45 mol⋅L^−1^ were injected into the flame with a syringe pusher (KD scientific, Holliston, MA, USA) at a constant speed equal to 3 mL⋅min^−1^ and sprayed by oxygen flow through the nozzle. The oxygen pressure drop across the nozzle (99.95% purity, NII KM, Moscow, Russia) was maintained at a level of 3 atm with a flow rate of 3 L⋅min^−1^. A flame of a mixture of methane (99.9% purity, NII KM, Moscow, Russia) and oxygen in a ratio of 1:2 was used for combustion ignition. Organic components were burned during the synthesis, and the reaction product was collected on a glass fiber GF/A filter (GE Whatman, Sigma-Aldrich, St. Louis, MO, USA) located 90 cm above the nozzle using a vacuum pump ISP 250 C (Anest Iwata, Yokohama, Japan), taken manually with a spatula from the filter surface and annealed in a muffle furnace in air at 500 °C for 24 h. Thus, materials based on TiO_2_ were obtained with a chromium content of χCr = [Cr]/([Cr] + [Ti]) = 0, 2, 4, 8, 10, 20, and 40 mol%.

Transmission electron microscopy studies were carried out on a high-resolution electron microscope JEOL JEM-2100 at 200 kV, as well as on a Tecnai Osiris electron microscope, (FEI, Hillsboro, OR, USA). Bright-field photographs of materials were obtained, including high-resolution images as well as electron diffraction patterns. The mapping of elements in the local areas of the samples was carried out using energy-dispersive X-ray spectroscopy.

The samples were studied by X-ray diffraction using a DRON-4-07 diffractometer (Burevestnik, St. Petersburg, Russia) using Cu K_α1+2_ radiation (λ = 1.5406 Å) in the range of 10–80° 2θ and a step of 0.1°. The phases present were identified using the ICDD PDF-2 database. Polycrystalline germanium was added to the samples as an internal standard. The sizes of the crystalline grains of the TiO_2_ oxide phases were estimated using the Scherrer formula. The unit cell parameters of the anatase and rutile phases were calculated using the quadratic formula for the tetragonal singony. The mass fractions of the anatase TiO_2_ (ICDD 21–1272) and rutile TiO_2_ (ICDD 21–1276) phases were determined by the Chang method using the corundum numbers RIR [89].

Raman spectra were recorded using an i-Raman Plus spectrometer (BW Tek, Plainsboro, NJ, USA) equipped with a BAC 151C microscope in the Raman frequency range of 80–1200 cm^−1^ with a resolution of 4 cm^−1^. A laser with a wavelength of 532 nm was used as a radiation source.

EPR spectra were recorded on an ELEXSYS-580 spectrometer (Bruker, Billerica, MA, USA) (X-band, sensitivity ~10^10^ spin⋅G^−1^) at an operating frequency of 9.5 GHz. Theoretical spectra simulated in the Easyspin program were used to confirm the qualitative and quantitative composition of spin centers in the material. Experimental spectra were obtained at 120 K.

The surface composition and charge state of the elements were studied by X-ray photoelectron spectroscopy (XPS). The spectra were recorded on an OMICRON ESCA+ (Scienta Omicron, Uppsala, Sweden) with an aluminum cathode equipped with a monochromatic X-ray source Al K_α_ XM1000 with an energy of hν = 1486.6 eV and a power of 252 W in a vacuum no lower than 10^−9^ mm of Hg. A CN-10 charge neutralizer with an emission current of 6 μA and a beam energy of 1 eV was used to avoid the appearance of charging effects. For each of the samples, a survey spectrum was obtained in the range of 1100–0 eV with a step of 0.5 eV. Spectra for elemental regions were recorded with a step of 0.1 eV. All spectra were accumulated at least three times and were corrected for the position of C_1s_ 285.00 eV. Quantitative analysis was carried out, taking into account the sensitivity coefficients of the elements and peak intensity values.

The specific surface area of the materials was measured by the method of low-temperature nitrogen adsorption, and the calculations were carried out according to the BET model. The measurements were carried out in a single-point mode on a Chemisorb 2750 instrument (Micromeritics, Norcross, GA, USA) equipped with a thermal conductivity detector. Surface preparation to remove unwanted adsorbed impurities included preliminary annealing in a flow of He (50 mL⋅min^−1^) at 300 °C for 1 h.

Gas sensors were manufactured by the deposition of a sensitive layer of synthesized materials on the surface of a corundum plate (2 mm × 2 mm × 0.15 mm) with platinum contacts for measuring resistance on one side and platinum heating elements on the other [24]. A paste with a concentration of approximately 200 mg/mL was prepared from the material powder and α-terpineol. This paste was deposited manually using a microspatula on the surface of the corundum plate (2 mm × 2 mm × 0.15 mm) with platinum contacts for measuring resistance on one side and the platinum heating element on the other. A layer of paste was applied so that the complete wetting of the chip surface was ensured. After each layer deposition, a ramped voltage (up to 5 V) with a duration of 120 s was applied to the heating element using an Agilent E3648A laboratory power supply unit (Agilent Technologies, Santa Clara, CA, USA). During such annealing, the binder component was removed. A total of 5–7 coats were applied in such a way as to completely cover the platinum contacts (see Figure S9). The obtained films were annealed at 500 °C for 12 h using the heating element of the sensor itself.

The sensor properties of the nanocrystalline materials based on TiO_2_ with a chromium content of 10, 20, and 40 at.%, as well as pure TiO_2_ and Cr_2_O_3_, were investigated in relation to a wide range of gasses and VOCs by the in situ measurement of the electrical conductivity of materials in a flow-through gas chamber in DC mode at 1 V voltage applied to the contacts for resistance measurements. Attested gas mixtures were used as gas sources. The electronic mass flow controllers (Bronkhorst, Nijverheidsstraat, The Netherlands) were used for gas mixing. The required humidity level was ensured by the mixing of two streams: dry air (χ(O_2_) = 20.95 vol%, RH = 0%) from a generator of clean air GClA-1.2-3.5 (Himelektronika, Moscow, Russia) and moist air (part of the flow is directed through a system of flasks with distilled water). Humidity values were recorded using a moisture meter IVTM-7 (Eksis, Zelenograd, Russia). The change in resistance was recorded after the change in gas flow from the gas mixture to clean air.

To investigate changes in sensor materials under measurement conditions, materials in a quartz cuvette were placed in a tubular furnace through which, at a certain temperature and humidity, air streams containing analyte vapors and pure air were alternately passed. The same frequency of changes in the gas atmosphere was used to simulate sensor measurements. Powder samples were taken from the cuvette at regular intervals and analyzed by X-ray diffraction and EPR spectroscopy.

## 4. Conclusions

The FSP technique allowed a facile, highly controlled and reproducible single-step synthesis of a series of nanocomposite materials in the system of p-conducting Cr-doped TiO_2_ and Cr_2_O_3_ with p-p heterojunction. The materials, which were obtained and described for the first time, demonstrated a three-orders-of-magnitude decrease in electrical resistance compared to pure TiO_2_ alongside with improved sensor response oxygenated VOCs at significantly lower working temperatures with a limited influence of humidity, which makes them potentially practically applicable for, e.g., medical diagnostics through exhaled air, although the cross-sensitivity to H_2_S should not be neglected in this regard. The formed p-p heterojunction is decisive for the observed value of the gas sensor response as it is controlling the free-electron transfer from the Cr-doped TiO_2_ phase to the surface-dispersed Cr_2_O_3_ during the gas-sensing process. Thorough materials’ examination with our set of methods revealed the presence of highly acidic Cr(VI) cations on the materials’ surface, which facilitates VOCs adsorption and conversion during the sensing process. The nanocomposite metal oxide materials take around a week to reach their stable gas-sensing performance as the slow processes of defect formation and sintering, unveiled by a combination of XRD and EPR techniques, are passing at an elevated working temperature. This nature of gas sensor properties improving over time makes it possible to shorten materials’ maturing prior to use by additional sintering at 500 °C or higher. The complex nature of the given study allows us to suggest that the system can possibly be further improved in terms of sensor response by the formation of p-p heterojunction between the nanocrystalline p-type Cr_2_O_3_ and only lightly Cr-doped TiO_2_ with a Cr content of around 2%, which demonstrates almost electrical insulating behavior due to the scarcity of charge carriers—either electrons or holes. Most likely, though, it will require some complication of the synthesis method.

## Figures and Tables

**Table 1:** XRD and BET characteristics of TiO_2_-Cr materials.

Material [a]	Anatase/ Rutile Ratio [b]	d_XRD_ [c] [nm]		S_BET_ [d] [m^2^/g]	d_BET_ [e] ± 2 [nm]	d_BET_/d_XRD_ [f]	*a* = *b* [Ȧ]		*c* [Ȧ]	
		A	R				A [g]	R [g]	A [g]	R [g]
TiO_2_	6.90	18 ± 0.5	17 ± 2	34 ± 2	46	2.5	3.7712	4.5806	9.4634	2.9441
TiO_2_-Cr-2	6.87	23 ± 0.5	24 ± 1	43 ± 3	36	1.6	3.7726	4.5840	9.4668	2.9445
TiO_2_-Cr-4	4.75	23 ± 1	21 ± 1	43 ± 3	36	1.6	3.7749	4.5881	9.4677	2.9453
TiO_2_-Cr-8	2.08	23 ± 1	21 ± 0.5	55 ± 4	28	1.3	3.7748	4.5884	9.4676	2.9456
TiO_2_-Cr-10	0.48	24 ± 2	20 ± 1	49 ± 3	30	1.4	3.7748	4.5888	9.4679	2.9456
TiO_2_-Cr-20	0.02	-	21 ± 2	50 ± 3	29	1.4	-	4.5890	-	2.9456
TiO_2_-Cr-40	0	-	13 ± 1	66 ± 5	21	1.6	-	4.5806	-	2.9441

[a] The value of the Cr mole fraction specified during synthesis is given. [b] The mass fractions ω of the anatase and rutile TiO_2_ phases were determined by the Chang method. [c] The crystal grain sizes of the TiO_2_ oxide phases were estimated using the Scherrer formula. [d] Specific surface area was determined by BET method. [e] The size of TiO_2_ agglomerates was determined from specific surface area. [f] Agglomeration degree. [g] The values were obtained by solving a system of equations using the quadratic form formula for tetragonal crystal.

**Table 2:** XPS characterization data.

Sample	χ_XPS_(Cr) [mol %]	Binding Energy [eV]								Ratio		s-o Splitting [eV]		
		Ti2p_3/2_	Ti2p_1/2_	O1s		Cr2p_3/2_		ΔTi^4+^-O_lat_ [eV]	ΔCr^3+^-O_lat_ [eV]	$\frac{{Cr}^{6+}}{{Cr}^{3+}}$	$\frac{O_{surf.}}{O_{latt.}}$			
		4+	3+	lat	surf *^a^*	3+	6+					Ti^4+^	Cr^3+^	Cr^6+^
TiO_2_	0	458.8	-	530.0	531.7	-	-	71.2	-	-	0.15	5.70	-	-
TiO_2_-Cr-10	9	458.5	459.4	529.9	531.4	577.0	579.4	71.4	47.1	0.65	0.92	5.85	9.69	8.95
TiO_2_-Cr-20	12	458.2	459.1	529.7	531.4	576.6	578.7	71.5	46.9	0.59	0.96	5.77	9.73	9.54
TiO_2_-Cr-40	16	458.3	459.2	529.9	531.1 532.3	576.7	578.5	71.6	46.8	0.59	0.93	5.73	9.81	10.17

*^a^* O-H and H_2_O surf., resp., for TiO_2_-Cr-40 material. Add. Inf.: ΔTi^3+^-O_lat_: 70.5, 70.8, and 70.7 [eV] for TiO_2_-Cr-10, TiO_2_-Cr-20, and TiO_2_-Cr-40, resp. ΔCr^6+^-O_lat_: 49.5, 49.0, and 48.6 [eV] for TiO_2_-Cr-10, TiO_2_-Cr-20, and TiO_2_-Cr-40, resp.

**Table 3:** Response and recovery times toward methanol (20 ppm) in humid air (RH = 60%).

At the Beginning of Long-Term Experiment			At the End of Experiment	
	Resp. time, sec	Rec. time, sec	Resp. time, sec	Rec. time, sec
TiO_2_	174 ± 3	292 ± 3	179 ± 3	287 ± 8
TiO_2_-Cr-10	220 ± 2	200 ± 1	224 ± 3	206 ± 6
TiO2-Cr-20	233 ± 4	186 ± 1	167 ± 7	193 ± 7
TiO_2_-Cr-40	222 ± 5	202 ± 1	162 ± 7	198 ± 6

## Data Availability

The data presented in this study are available on request from the corresponding author.

## Supplementary Materials

The supporting information can be downloaded at: https://www.mdpi.com/article/10.3390/ijms26020499/s1.
