# Supplementary material for: Cr-Doped Nanocrystalline TiO2-Cr2O3 Nanocomposites with p-p Heterojunction as a Stable Gas-Sensitive Material
Source: Int J Mol Sci. 2025 Jan 9;26(2):499. doi: 10.3390/ijms26020499 (PMC11765019; doi:10.3390/ijms26020499)
Supplement: Supplementary file 1 [file ijms-26-00499-s001.zip › ijms-3355200-supplementary.pdf]

International Journal of *Molecular Sciences*

MDPI

Article

Cr-Doped Nanocrystalline TiO_2-Cr_2O_3 nanocomposites with p-p heterojunction as a Stable Gas Sensitive Material

Dmitriy Kuranov [1]*, Elizaveta Konstantinova [2], Anastasia Grebenkina [1], Alina Sagitova [1], Vadim Platonov [1], Sergey Polomoshnov [3], Marina Rumyantseva [1] and Valeriy Krivetskiy [1,4]*

[1] Chemistry Department, Lomonosov Moscow State University, 119991 Moscow, Russia; kuranov.mitya@mail.ru (D.K.); nastya.greb@yandex.ru (A.G.); sagitova@inorg.chem.msu.ru (A.S.); agnes1992@yandex.ru (V.P.); vkrivetsky@inorg.chem.msu.ru (V.K.)
[2] Physics Department, Lomonosov Moscow State University, 119991 Moscow, Russia; liza35@mail.ru
[3] National Research University of Electronic Technology 124498 Zelenograd, Moscow, Russia psa@org.miet.ru
[4] Scientific-Manufacturing Complex Technological Centre, 124498 Moscow, Russia

*Correspondence: kuranov.mitya@mail.ru, vkrivetsky@inorg.chem.msu.ru

Abstract: Nanocrystalline TiO_2 is a perspective semiconductor gas sensing material due to its long-term stability of performance, but limited in application because of high electrical resistance. In this paper a gas sensing nanocomposite material with p-p heterojunction is introduced based on p-conducting Cr-doped TiO_2 in combination with p-conducting Cr_2O_3. Materials were synthesized via single step flame spray pyrolysis (FSP) technique and comprehensively studied by XRD, BET, TEM, EDX, XPS, EPR and Raman spectroscopy. Gas sensor performance in DC mode was studied towards a number of gases (H_2, CO, CH_4, NO_2, H_2S, NH_3) as well as volatile organic compounds (VOCs) (acetone, methanol and formaldehyde) in dry and humid conditions. Long-term stability of obtained materials gas sensor performance was evaluated alongside with *ex situ* study of structural evolution. High sensitivity towards oxygenated VOCs and lower detection limit below ppm level with limited influence of humidity was shown. Long-term gas sensor performance stability of obtained materials and its connection to the defect structure of doped TiO_2 is demonstrated.

Keywords: titanium dioxide, doping, pyrolysis, chromium, semiconductors, stability

1. Supplementary figures

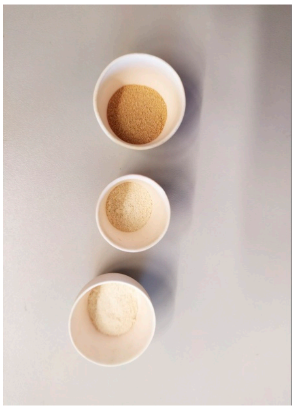

Figure S1. The photographic images of TiO_2 and TiO_2-Cr powders after post-synthetic annealing (TiO_2, TiO_2-Cr-8, TiO_2-Cr-40 materials shown).

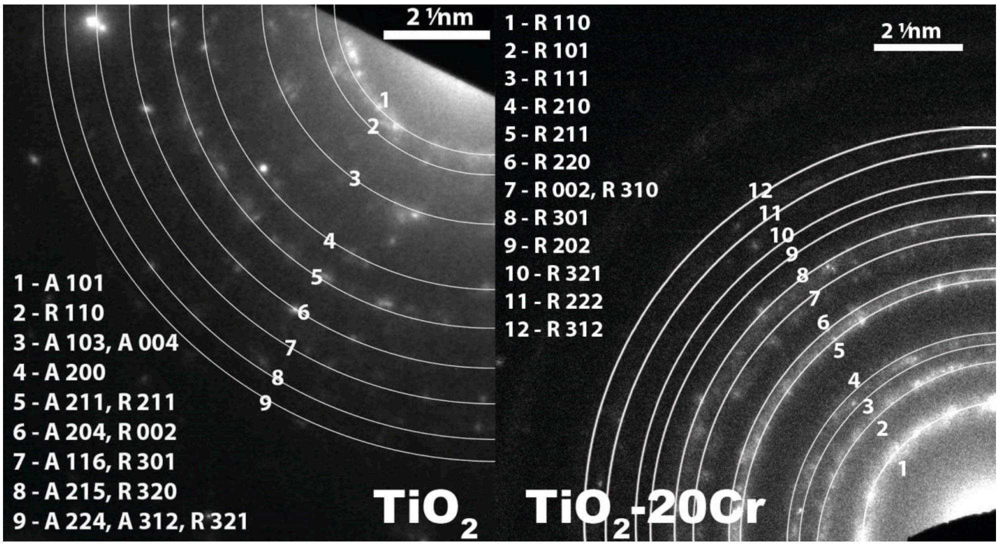

Figure S2. Indexed electron diffraction patterns for TiO_2 and TiO_2-Cr-20 materials.

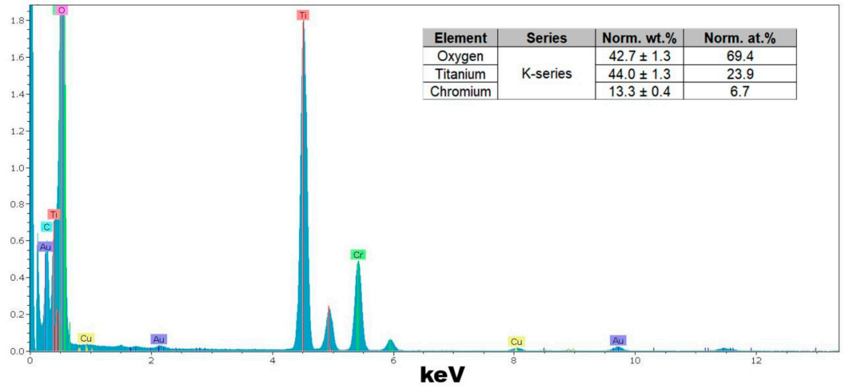

Figure S3. Energy-dispersive X-ray data for TiO_2-Cr-20 (pointed at the center of the particle).

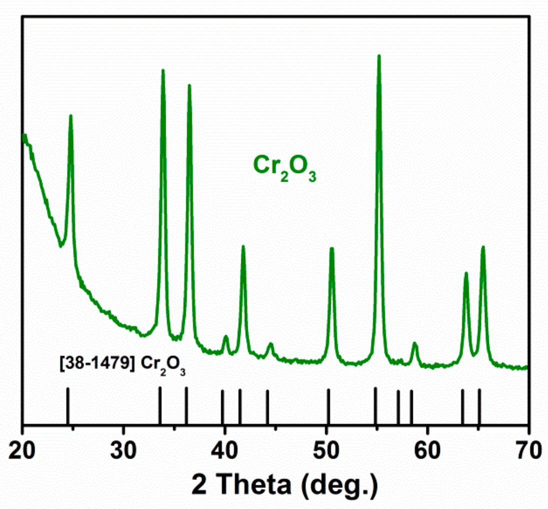

Figure S4. The X-ray diffraction pattern of pure Cr_2O_3.

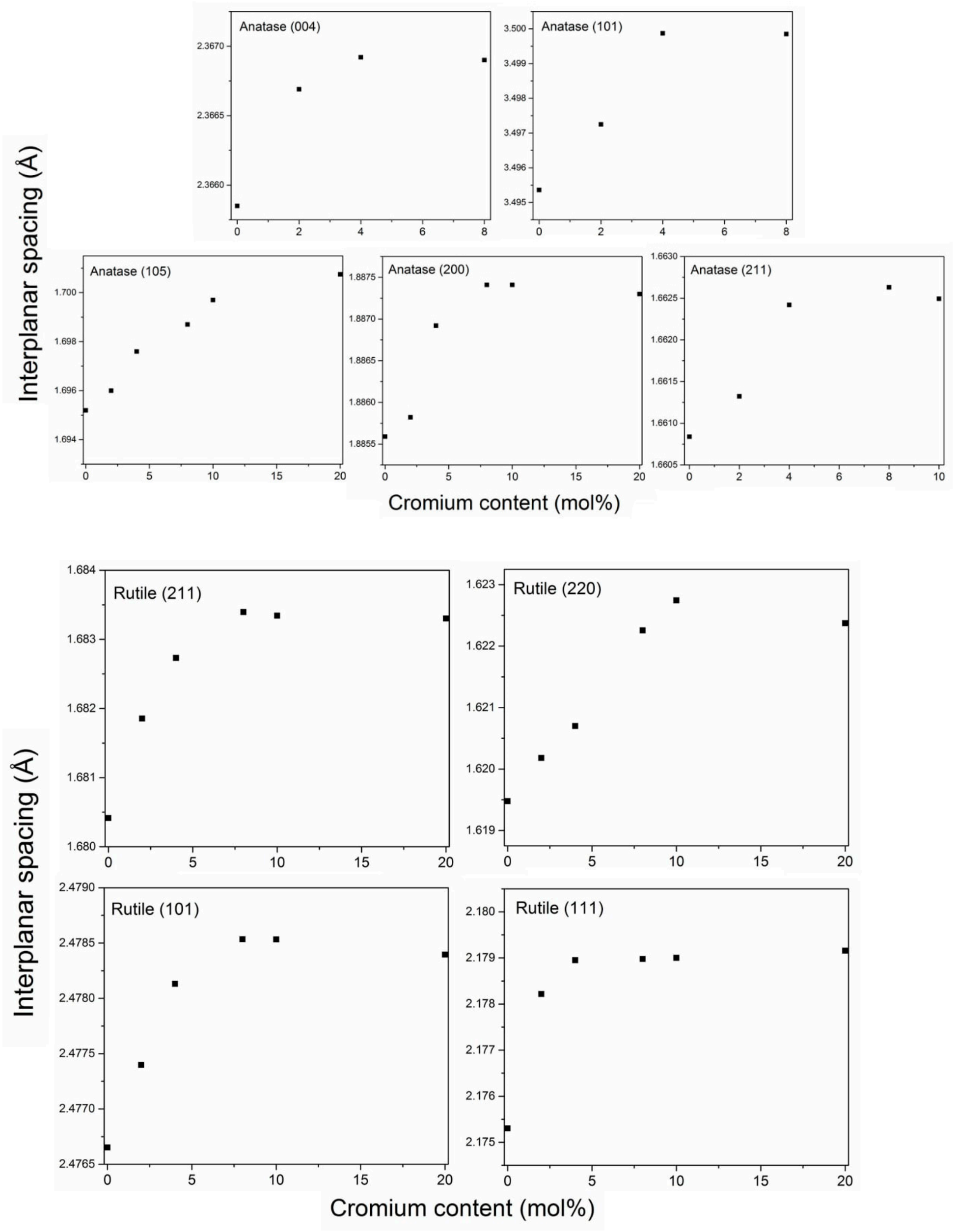

Figure S5. Changes in the values of some interplanar spaces during doping (anatase and rutile phases).

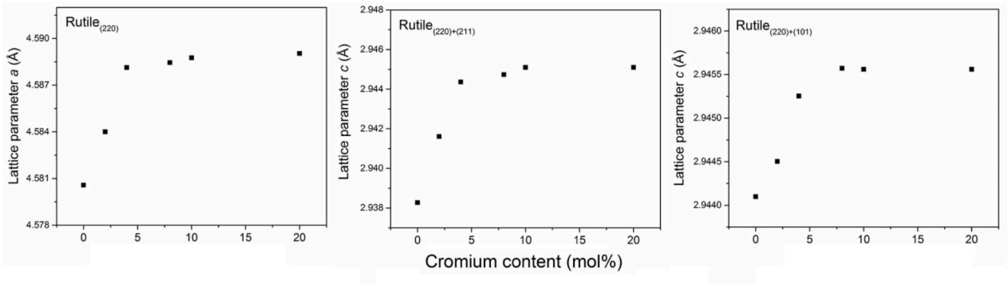

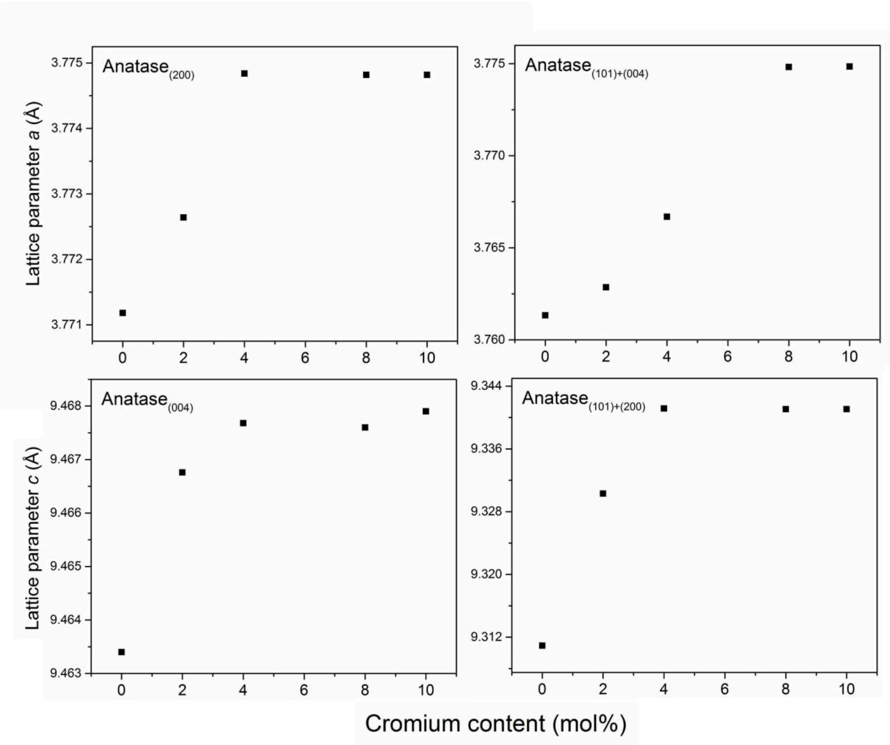

Figure S6. The obtained values of the unit cell parameters of doped samples (rutile and anatase phase).

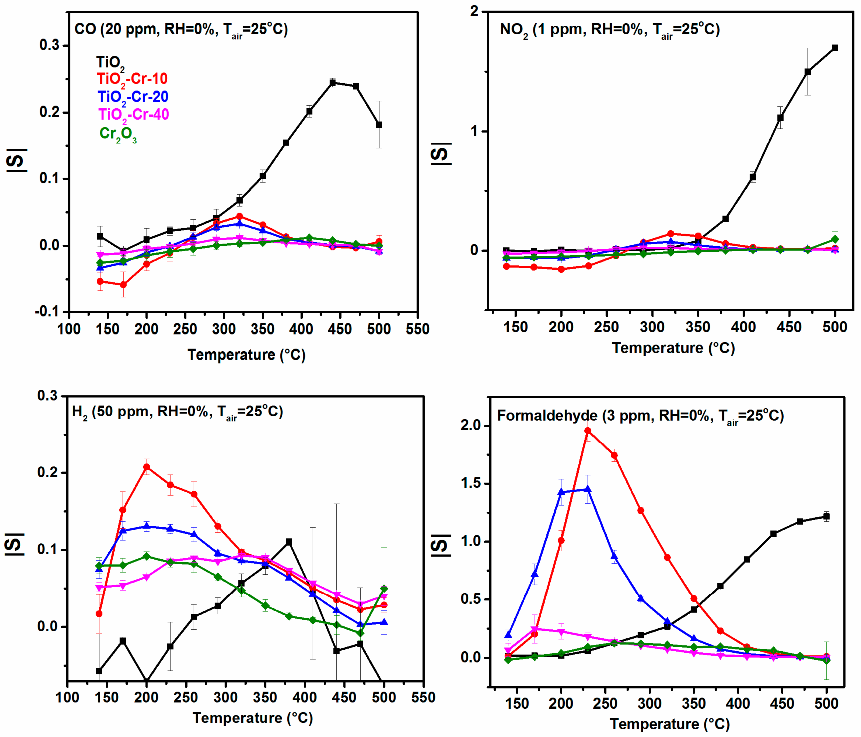

Figure S7. Temperature dependencies of the sensor signal of all materials. The modulus of sensor response is given for the sake of the easier comparison, while all Cr-containing materials behave as p-type semiconductors and pure TiO_2 has shown n-type behavior.

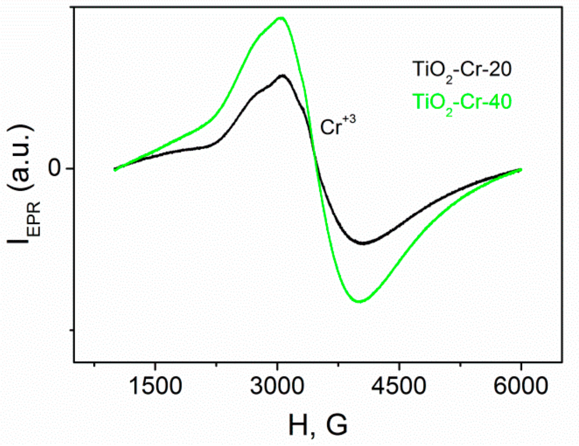

Figure S8. EPR spectra of TiO_2-Cr-20 and TiO_2-Cr-40.

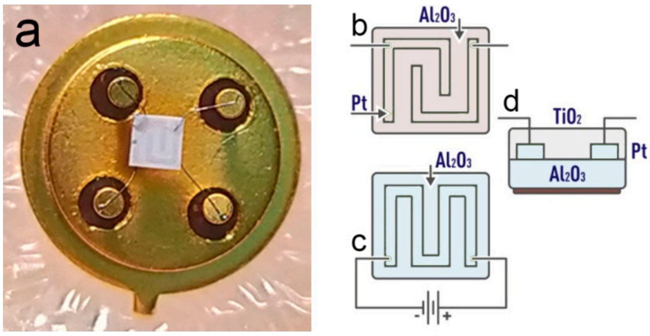

Figure S9. Optical image of the sensor (a) and a schematic representation of the sensitive element (top: b, back: c, side: d). Reprinted with permission from [1].

References

1. Kuranov, D.; Platonov, V.; Konstantinova, E.; Grebenkina, A.; Rumyantseva, M.; Polomoshnov, S.; Krivetskiy, V. Gas Sensing with Nb(V) Doped Nanocrystalline TiO2: Sensitivity and Long-Term Stability Study. *Sensors Actuators B Chem.* **2023**, *396*, 134618, doi:10.1016/j.snb.2023.134618.
